# Technology platform development for targeted plasma metabolites in human heart failure

**DOI:** 10.1186/1559-0275-10-7

**Published:** 2013-07-04

**Authors:** CY X’avia Chan, Anjum A Khan, JH Howard Choi, CM Dominic Ng, Martin Cadeiras, Mario Deng, Peipei Ping

**Affiliations:** 1 NHLBI Proteomics Center at UCLA, Departments of Physiology and Medicine, David Geffen School of Medicine, University of California at Los Angeles, Los Angeles, CA 90095, USA; 2 Thermo Fisher Scientific, San Jose, CA 95134, USA; 3 Ronald Reagan UCLA Medical Center, UCLA Medical Center, Los Angeles, CA 90095, USA

**Keywords:** Heart disease, Targeted human plasma metabolomics, Sample preparation

## Abstract

**Background:**

Heart failure is a multifactorial disease associated with staggeringly high morbidity and motility. Recently, alterations of multiple metabolites have been implicated in heart failure; however, the lack of an effective technology platform to assess these metabolites has limited our understanding on how they contribute to this disease phenotype. We have successfully developed a new workflow combining specific sample preparation with tandem mass spectrometry that enables us to extract most of the targeted metabolites. 19 metabolites were chosen ascribing to their biological relevance to heart failure, including extracellular matrix remodeling, inflammation, insulin resistance, renal dysfunction, and cardioprotection against ischemic injury.

**Results:**

In this report, we systematically engineered, optimized and refined a protocol applicable to human plasma samples; this study contributes to the methodology development with respect to deproteinization, incubation, reconstitution, and detection with mass spectrometry. The deproteinization step was optimized with 20% methanol/ethanol at a plasma:solvent ratio of 1:3. Subsequently, an incubation step was implemented which remarkably enhanced the metabolite signals and the number of metabolite peaks detected by mass spectrometry in both positive and negative modes. With respect to the step of reconstitution, 0.1% formic acid was designated as the reconstitution solvent vs. 6.5 mM ammonium bicarbonate, based on the comparable number of metabolite peaks detected in both solvents, and yet the signal detected in the former was higher. By adapting this finalized protocol, we were able to retrieve 13 out of 19 targeted metabolites from human plasma.

**Conclusions:**

We have successfully devised a simple albeit effective workflow for the targeted plasma metabolites relevant to human heart failure. This will be employed in tandem with high throughput liquid chromatography mass spectrometry platform to validate and characterize these potential metabolic biomarkers for diagnostic and therapeutic development of heart failure patients.

## Background

Heart failure refers to the cardiac malfunction in pumping sufficient amount of blood to meet the metabolic demand of the body. To date, there are over 6.6 million heart failure patients in the U.S. [1]. In spite of the integration of various medical regiments, such as ACE-inhibitors, β-blockers, the morbidity and mortality of heart failure patients remain staggeringly high, in which more than 80% of heart failure patients have to be hospitalized [2]; 50% of which die within 5 years of the diagnosis [3,4]. This dramatic situation places an enormous burden on both the health care sector as well as the economy. Furthermore, the existing screening tests adapted to examine various classic cardiovascular risk factors, such as hypertension, hyperlipidemia, smoking, and diabetes…etc. cannot fully delineate and precisely evaluate the inter-individual variation [5]. This limitation urges an expanded and comprehensive diagnostic platform to achieve an accurate assessment of the disease stage, as well as the subsequent complementary treatments for heart failure patients.

Heart failure is a multifactorial and multigenic disease, in which several biological processes have shown to be critical, including extracellular matrix remodeling, energy metabolism, and inflammation. In the aspect of extracellular matrix remodeling, ischemic heart failure is characterized by massive fibrous tissue formation at the site of myocardial infarction [6,7] and its vicinity. This uncontrollable extracellular matrix accumulation leads to myocardial stiffness, and hence the cardiac contractility is impaired [8-13]. In the aspect of energy metabolism, malfunction was reported in heart failure patients, whom encountered severe energy deprivation, accompanied by muscular fatigue and low exercise intolerance [14-18]. In the context of myocardial inflammation, its pathophysiological relevance to heart failure has been recognized since 1669 [19]. Chronic heart failure patients displayed episodes of systemic inflammation, as evident by the increased circulating cytokines [20,21]. In view of the aforementioned pathological phenotypes of heart failure and their relevant biology in metabolism, we designed and implemented a metabolomic approach in an effort to profile metabolites, such as lipids, amino acids, and sugars, whose functions are to maintain and orchestrate the normal biological processes and phenotype of an organism [22-31].

Metabolomics has emerged as an effective technical strategy for the development of efficient diagnostic markers and therapeutic interventions [32-34]. Previously, we have adapted an approach combining proteomic and nuclear magnetic resonance spectroscopy-based metabolomics to identify key proteins and cardiac energy metabolites involved in cardioprotection during ischemia/reperfusion injury [35]. In this investigation, we initiated an effort to establish a mass spectrometry-based metabolomic analytical platform to characterize and to validate potential metabolic markers in heart failure, particularly in plasma due to its less-invasiveness nature compared to biopsy [36]; also its circulating nature enables us to obtain a temporal physiologic status of the patients [37]. 19 metabolites were chosen based on their demonstrated functions in the relevant biological processes to heart failure, specifically participating in the regulation of extracellular matrix remodeling [38], energy metabolism [39-41], inflammation [42], insulin resistance [43], renal dysfunction [44], and cardioprotection [45].

Despite the development of new technologies, to date, no protocols are available to extract all targeted metabolites in a singular sample preparation due to their broad dynamic range and their diverse chemical properties. With the limited sample volume from the patients, we encountered technical challenges to profile these metabolites in parallel in clinical studies. It is pivotal to devise a simple yet effective workflow that enables us to extract most, if not all, of the targeted metabolites of interest. Accordingly, we organized a focused effort in this study to develop and to optimize a platform enabling the characterization of multiple metabolites in one single analysis. Prior to proceeding with the clinical sample analysis, it is crucial to ensure the efficiency of the workflow and to resolve any technical shortfalls. Therefore, we systematically engineered, optimized and refined a protocol applicable to human plasma.

## Results and discussion

**Figure 1:**
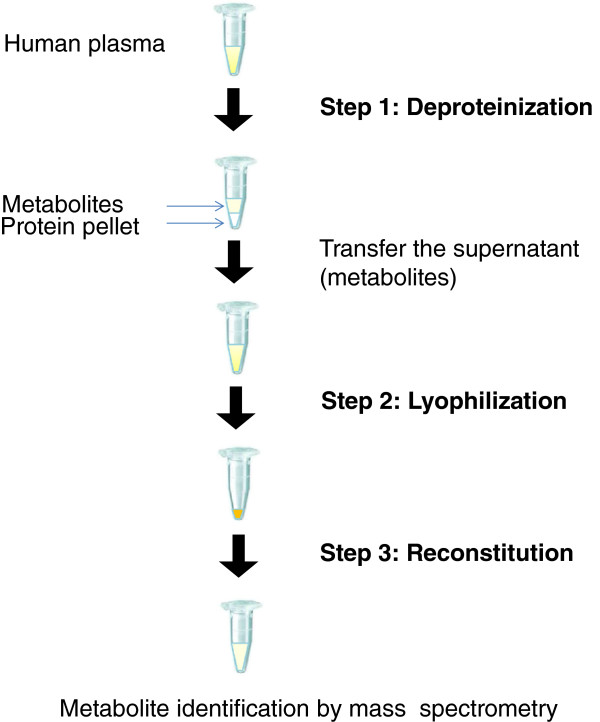
**An overview of existing workflow for sample preparation in the studies of human plasma metabolomics.** Human plasma is first deproteinized with organic solvent, in which proteins are precipitated (step 1). The metabolites in the supernatant are then subjected to lyophilization (step 2) and reconstitution for subsequent mass spectrometric analyses (step 3).

The sample preparation workflow was adjusted and refined in the levels of deproteinization, incubation, and reconstitution; the general pipeline is illustrated in Figure 1. In brief, sample is first deproteinized with organic solvent, in which the proteins are precipitated, while the metabolites remain in the supernatant. After which, the metabolites in the supernatant are subjected to lyophilization. The pellet as result is reconstituted in a solvent that is susceptible to the downstream analytical platform of choice. In this report, we systematically optimized and validated each of these steps, and have successfully established a finalized protocol which enabled us to retrieve 13 out of 19 of our targeted metabolites, as identified by mass spectrometry.

### Optimization of deproteinization

**Figure 2:**
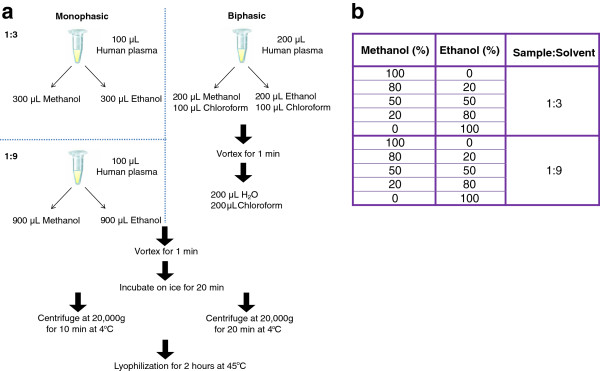
**Optimization of deproteinization. (a)** In the step of monophasic deproteinization, two samples containing either plasma:solvent ratios of 1:3 or 1:9 were examined. In the condition of a 1:3 ratio, 300 μL of either methanol or ethanol was added to 100 μL of plasma; whereas in the setting of a 1:9 ratio, 900 μL of methanol or ethanol was added to 100 μL of plasma. In the aspect of biphasic deproteinization, a two-step solvent addition was introduced, starting with an addition of 100 μL chloroform with 200 μL of either methanol or ethanol to 200 μL of plasma, followed by an addition of 400 μL 50% chloroform/H_2_O. Metabolites extracted via both mono- and biphasic methods were subjected to a 1-minute vortexing, and a 20-minute incubation on ice. After centrifugation, the metabolites in the supernatants were subsequently lyophilized at 45°C for 2 hours. **(b)** To further optimize the step of monophasic deproteinization, various combinations of methanol/ethanol were prepared, and added to the plasma at a ratio of either 1:3 or 1:9 (plasma:solvent).

Deproteinization is a crucial step that significantly impacts the yield and types of metabolites to be extracted. Conventionally, there are two types of extraction methods: monophasic and biphasic. The former is commonly adapted because of its simplicity, in which a certain proportion of organic solvent is added to the sample in a single step [46,47]. To assess the efficiency of the two extraction methods, four sets of plasma samples were prepared. In monophasic extraction, ethanol or methanol was added at a ratio of 1:3 or 1:9 (plasma:solvent), respectively. In biphasic extraction, chloroform with either methanol or ethanol was added; followed by 50% chloroform. After centrifugation, the hydrophilic and hydrophobic metabolites were separated and collected (Figure 2a). In Table 1 and in Additional file 1, the monophasic approach offered the identification of a greater number of metabolites. Therefore, we elected the monophasic deproteinization for our study. As illustrated in Table 1, extraction of plasma samples by either 100% of methanol or 100% of ethanol generated comparable panel of metabolites. To systematically refine the monophasic deproteinization that best suitable for our targeted metabolites, different combinations of methanol/ethanol (100%/0%, 80%/20%, 50%/50%, 20%/80%, and 0%/100%) were examined; they were added to the plasma sample at a ratio of plasma:solvent volume either 1:3 or 1:9 (Figure 2b). As summarized in Table 2 and in Additional file 2, maximum number of targeted metabolites were identified via the extraction step containing 20% methanol/ethanol with a plasma:solvent ratio of 1:3.

**Table 1:** Targeted human plasma metabolites identified in monophasic and biphasic deproteinizations

**Metabolite name**	**Accesion #**	**Monophasic**				**Biphasic**	
		**1:3 Methanol**	**1:3 Ethanol**	**1:9 Methanol**	**1:9 Ethanol**	**Methanol/Chloroform**	**Ethanol/Chloroform**
Hexanoic acid	HMDB00535	x				x	x
Succinate	HMDB00254					x	
Hypoxanthine	HMDB00157			x	x		
L-lysine	HMDB00182		x				
Uric acid	HMDB00289	x					
L-Arginine	HMDB00517		x				
Dodecanoic acid	HMDB00638	x	x			x	x
Palmitatic acid	HMDB00220	x	x	x	x	x	x
Linoleatic acid	HMDB00673	x	x	x	x	x	x
Stearic acid	HMDB00827	x	x	x	x	x	x

**Table 2:** Further in-depth optimization of monophasic deproteinization using different combinations of methanol/ethanol mix and plasma:solvent ratios of 1:3 and 1:9, respectively

		**1:3(Plasma:Solvent)**					**1:9(Plasma:Solvent)**				
**Metabolite name**	**Accesion #**	**100% Methanol**	**80% Methanol/Ethanol**	**50% Methanol/Ethanol**	**20% Methanol/Ethanol**	**100% Ethanol**	**100% Methanol**	**80% Methanol/Ethanol**	**50% Methanol/Ethanol**	**20% Methanol/Ethanol**	**100% Ethanol**
Hexanoic acid	HMDB00535	x	x	x	x		x		x		x
Succinate	HMDB00254			x	x	x	x		x	x	x
1-Methylhistamine	HMDB00898				x						
Trans −4-Hydrxy-L-proline	HMDB00725	x		x	x	x					
Creatine	HMDB00064	x		x	x						
L-Malate	HMDB00156	x			x	x					x
Hypoxathine	HMDB00157	x		x	x	x	x		x		x
a-Ketoglutaric acid	HMDB00208										
L-lysine	HMDB00182										
Uric acid	HMDB00289										
L-Arginine	HMDB00517										
L-Tyrosine	HMDB00158										
Indole-3-propionate	HMDB02302										
Citric acid	HMDB00094										
Dodecanoic acid	HMDB00638	x	x	x	x		x	x	x		x
Palmitatic acid	HMDB00220	x	x	x	x		x	x	x		x
Linoleatic acid	HMDB00673	x		x	x		x		x		x
Trans-Vaccenic acid	HMDB03231	x	x	x			x				x
Stearic acid	HMDB00827	x	x	x	x		x	x	x		x
# Targeted Meabolites		11	5	11	13	9	8	3	7	1	9

### The implementation of an incubation step improves the metabolite yield

**Figure 3:**
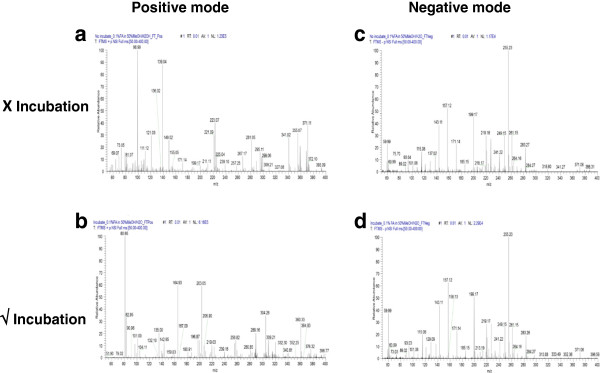
**Implementation of an incubation step. (a)** In the positive mode, the signal detected without an incubation step was 1.23E5. **(b)** This signal was remarkably increased to 6.18E5 with an additional 4 peaks detected amid the incorporation of an incubation step. **(c)** In the negative mode, the signal detected without the incubation step was 1.17E4. **(d)** This signal was enhanced to 2.29E4 with an additional 88 peaks detected amid the implementation of an incubation step.

According to the generic metabolite preparation protocol, once the sample is deproteinized with organic solvent, such as methanol, it will subsequently be subjected to lyophilization. We examined if an additional 20-minute incubation step on ice before lyophilization would enhance the metabolite yield. In this regard, two sets of plasma samples were prepared and deproteinized with methanol in a ratio of 1:3 (plasma:methanol). After which, one set was directly subjected to lyophilization; whereas the other was allowed to incubate on ice for 20 minutes prior to lyophilization. Metabolites derived from both sets were then reconstituted in 0.1% formic acid/50% methanol and subjected to a static nanoelectrospray ionization coupled to an LTQ orbitrap XL hybrid fourier transform mass spectrometer (FTMS). Mass spectra were acquired in both positive and negative modes. In the additional step with a 20-minute incubation on ice, there was a 5-fold increase in signal intensity which led to an additional 4 peaks detected in the positive mode; whereas in the negative mode, there was a 2-fold increase in signal which resulted the detection of an additional 88 peaks (Figure 3).

### Selection of a reconstitution solvent

**Figure 4:**
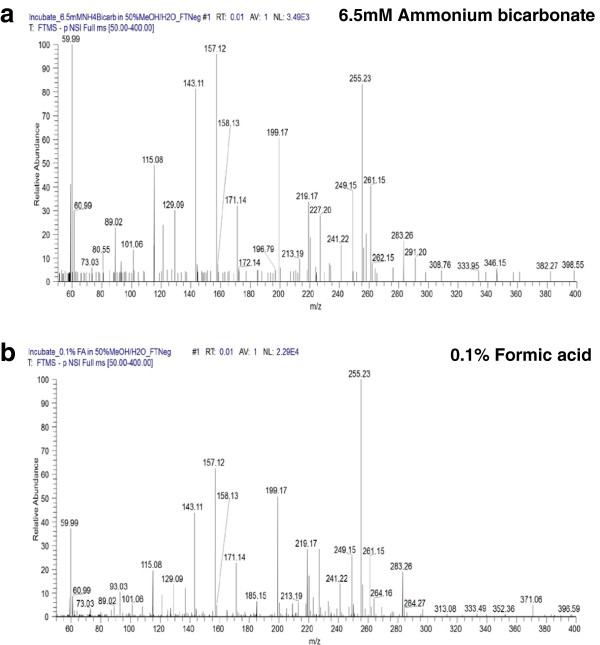
**Selection of a reconstitution solvent. (a)** In the setting of 6.5 mM ammonium bicarbonate, the signal intensity was recorded as 3.49E3. **(b)** In the setting of 0.1% formic acid, the signal was enhanced to 2.29E4, with an additional 108 peaks detected. Therefore, 0.1% formic acid was elected as the reconstitution solvent.

Previous study by Evans and colleagues reported that 0.1% formic acid favored formation of the positive ions, but suppressed the negative ions [48]. In this regard, ammonium bicarbonate was recommended as the reconstitution solvent in negative mode. Nonetheless, adapting two different reconstitution solvent involves an additional partition step to divide the plasma sample equally into two portions: one reconstituted in 0.1% formic acid for the positive mode; the other in ammonium bicarbonate for the negative mode. This extra partition step may introduce downstream quantitative variation due to the inevitable experimental error. Furthermore, during liquid chromatography-tandem mass spectrometry (LC-MS/MS) analysis, two different sets of LC buffer have to be prepared for both positive and negative modes; therefore an additional conditioning time is required upon switching the buffers. As a consequence, this process will attenuate the high throughput capability of LC-MS/MS analysis. To evaluate the outcome of adapting ammonium bicarbonate vs. 0.1% formic acid as the reconstitution buffer in the negative mode, two sets of plasma samples were deproteinized and lyophilized as aforementioned. One set was reconstituted in 0.1% formic acid, whereas the other in 6.5 mM ammonium bicarbonate, both of which were then subjected to mass spectrometric analysis in the negative mode. As illustrated in Figure 4, 0.1% formic acid yielded greater signal than ammonium bicarbonate (2.29E4 vs. 3.49E3, respectively). Specifically, this led to the detection of an additional 108 metabolite peaks. Therefore, 0.1% formic acid was chosen as the reconstitution solvent.

Adapting the workflow we devised, we were able to retrieve most of our targeted metabolites, except L-lysine, uric acid, and citric acid. This result may be due to the signal suppression occurred during the direct infusion, which could be circumvented by the incorporation of liquid chromatographic fractionation prior to mass spectrometric analysis.

## Conclusions

**Figure 5:**
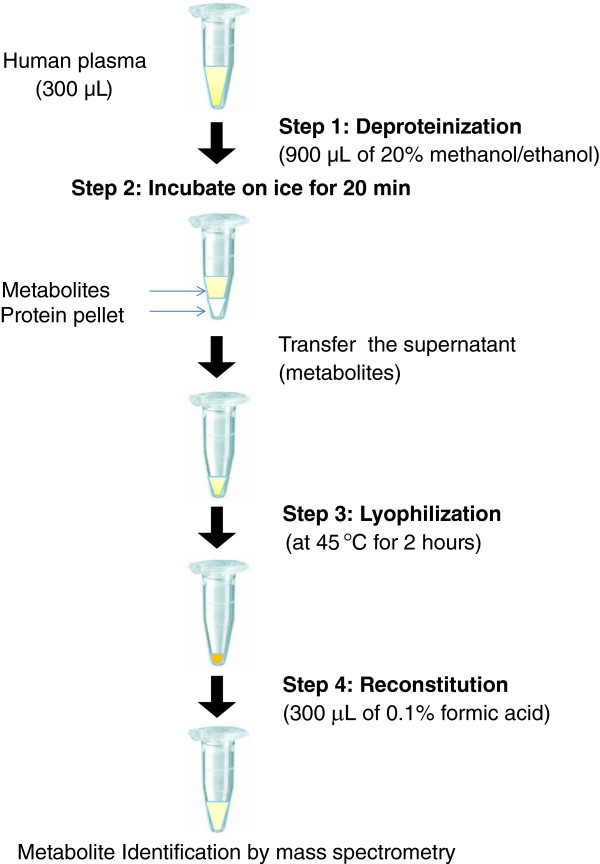
**Our recommended workflow for sample preparation in the studies of targeted human plasma metabolites.** 300 μL of human plasma was first deproteinized by 900 μL of 20% methanol/ethanol (step 1); then was incubated on ice for 20 minutes (step 2). After which, the metabolites in the supernatant were obtained and lyophilized at 45°C for 2 hours (step 3). The resulting pellet was subsequently reconstituted with 300 μL of 0.1% formic acid for mass spectrometric analyses (step 4).

In summary, an optimized sample preparation and workflow for targeted human plasma metabolites has been devised and outlined in Figure 5. This newly developed platform offers a simple albeit effective way to extract most (13 out of 19) of our targeted metabolites. This workflow in conjunction with LC-MS/MS will enable us to establish a high throughput metabolomic platform to characterize and validate these targeted metabolites as potential biomarker in human heart failure.

## Methods

### Chemicals

Methanol, ethanol, chloroform, and water were purchased from J.T. Baker (New Jersey, USA). Formic acid and ammonium bicarbonate were from Sigma (St. Louis, USA).

### Sample preparation for human plasma metabolites

Human whole blood was purchased from Biological Specialty Corporation (Colmar, USA). The whole blood was first diluted with PBS in 1:1, followed by a 30 minute-centrifugation at 700 g at room temperature. Plasma at the top layer was then obtained.

Monophasic and biphasic deproteinizations of plasma were depicted in Figure 2a and b. In brief, in the step of monophasic deproteinization, various combinations of methanol/ethanol (100%/0%; 80%/20%; 50%/50%; 20%/80%; and 0%/100%) were added to 100 μL of plasma at a ratio of either 1:3 or 1:9 (plasma:solvent). Subsequently, sample was vortexed for 1-minute and incubated on ice for 20 minutes. After centrifugation at 20,000 g for 10 minutes at 4°C, the supernatant was lyophilized at 45°C for 2 hours. In the step of biphasic deproteinization, 200 μL of plasma was mixed with 100 μL of chloroform and 200 μL of methanol or ethanol. After which, it was vortexed for 1 minute. 200 μL of water and 200 μL of chloroform were then added. After 1 minute of vortexing and 20 minutes of centrifugation at 20,000 g at 4°C, hydrophilic and hydrophobic metabolites at the top and bottom layers, respectively, were collected and lyophilized at 45°C for 2 hours. The lyophilized pellets as result were either reconstituted in equal initial plasma volume of 0.1% formic acid/H_2_O or 6.5 mM ammonium bicarbonate/H_2_O prior to mass spectrometric analysis.

### Mass spectra acquisition

20 μL of the reconstituted metabolite extract was loaded onto a nanoelectrospray tip (New Objective, Woburn, USA), and subjected to Thermo Scientific LTQ orbitrap XL hybrid FTMS for mass spectra acquisition. For ionization source parameters, capillary temperature was set at 275°C; while source and tube lens voltages were set at +/−2.2 kV and +/−130 V, respectively. FT full MS scan was acquired at 30,000 resolution with m/z range of 50–400 Th; whereas linear ion trap (LIT) MS/MS scan was obtained via collision induced dissociation (CID) with a normal m/z range of 50–400 Th; isolation width of 1 Th; and normalized collision energy of 35. All mass spectra were acquired in both positive and negative modes.

### Metabolite identification

All MS/MS spectra were searched against the Human Metabolome Database [49]. The search parameters were set as follow: parent ion mass tolerance, ±0.01; fragment ion m/z tolerance, ±0.1; CID energy, all. The outcome of this putative identification was further validated by the fragmentation pattern of the respective standards of the same set of metabolites (Additional file 3).

## Abbreviations

CID: Collision induced dissociation; FTMS: Fourier transform mass spectrometer; LC-MS/MS: Liquid chromatography-tandem mass spectrometry; LIT: Linear ion trap.

## Competing interests

The authors declare that they have no competing interests.

## Authors’ contributions

CY designed and performed all the experiments in this study, as well as the manuscript writing; AK and CM contributed their expertise on the instrumentation of mass spectrometry. JH provided bioinformatics knowledge on the identification of metabolites. MC and MD provided expertise in the human plasma sample processing. PP directed the NHLBI facility and oversaw the study. All authors read and approved the final manuscript.

## Supplementary Material

Additional file 1 Summary notes: This file summarized the identifications of targeted human plasma metabolites in the following deproteinization conditions: (1) Monophasic deproteinization using plasma: methanol (1:3); (2) monophasic deproteinization using plasma:ethanol (1:3); (3) monophasic deproteinization using plasma: methanol (1:9); (4) monophasic deproteinization using plasma: ethanol (1:9); (5) biphasic deproteinization using methanol and chloroform; and (6) biphasic deproteinization using ethanol and chloroform. Click here for file

Additional file 2 Summary notes: This file summarized the identifications of targeted human plasma metabolites in the following monophasic deproteinization conditions: (1) Plasma:methanol (1:3); (2) plasma: 80% methanol/ethanol (1:3); (3) plasma: 50% methanol/ethanol (1:3); (4) plasma: 20% methanol/ethanol (1:3); (5) plasma: ethanol (1:3); (6) plasma: methanol (1:9); (7) plasma: 80% methanol/ethanol (1:9); (8) plasma: 50% methanol/ethanol (1:9); (9) plasma: 20% methanol/ethanol (1:9); and (10) plasma: ethanol (1:9). Click here for file

Additional file 3 **Summary notes: This file illustrated the MS/MS spectra acquired from the human plasma metabolites extracted via the monophasic deproteinization using 20% methanol/ethanol.** The outcome of these putative identifications were further validated by the fragmentation pattern of the respective standards of the same set of metabolites. Click here for file
